# The COVID-19 pandemic related stress and the associated factors among the healthcare workers in Kota Setar District Health Office, Malaysia

**DOI:** 10.1371/journal.pone.0301469

**Published:** 2024-05-23

**Authors:** Mohd Faiz Itam, Halimatus Sakdiah Minhat, Anita Abd Rahman, Mohd Zukri Ibrahim, Shareh Azizan Shareh Ali, Ahmad Hanis Shuhaimi

**Affiliations:** 1 Faculty of Medicine and Health Sciences, Department of Community Health, Universiti Putra Malaysia, Serdang, Malaysia; 2 Malaysian Research Institute on Ageing, Universiti Putra Malaysia, Serdang, Selangor, Malaysia; 3 Kubang Pasu Health District Office, Jitra, Kedah, Malaysia; 4 Kota Setar Health District Office, Alor Setar, Kedah, Malaysia; National Cheng Kung University College of Medicine, TAIWAN

## Abstract

**Background:**

The COVID-19 pandemic is the most significant worldwide health catastrophe, with massive impacts observed particularly among the healthcare workers. Stress among healthcare workers is a significant and pervasive issue that can have profound implications for both the well-being of healthcare professionals and the quality of patient care. This study aimed to determine the prevalence of stress related to the COVID-19 pandemic and the associated factors among the healthcare workers (HCWs).

**Methods:**

A retrospective cross-sectional study was conducted involving 533 HCWs in Kota Setar District Health Office. Related data was collected between January and June 2021. Stress was measured using the Depression Anxiety Depression Scale 21 (DASS-21). Other variables included in this study were sociodemographic and employment factors. The associated factors and predictors were determined by employing chi-square test and multivariate logistic models.

**Results:**

COVID-19 related stress was reported at 10.5%. HCWs who work at the district health offices and those with degree or higher qualifications had 2.3 (AOR = 2.310, 95% CI: 1.177–4.535) and 3 (AOR = 2.899, 95% CI: 1.613–5.211) higher odds of experiencing stress compared to those working in the clinics and had lower qualifications (diploma or less).

**Conclusions:**

The mental wellbeing of the HCWs participated in this study had been affected negatively by the COVID-19 pandemic, resulting in one in 10^th^ of the HCWs were experiencing stress during the COVID-19 pandemic, with higher risk observed among those working at the district health office and HCWs with higher qualifications or ranking. This is expected since COVID-19 was a new and unprecedented outbreak associated with massive number of mortalities that requires active contact tracing and surveillance which commonly conducted at district health office level. Active intervention needed to cope with the overwhelming stress and working condition to ensure effective rehabilitation are in place and quality of work were not jeopardized.

## Introduction

The coronavirus disease 2019 (COVID-19) pandemic has negatively impacted public health systems worldwide and created anxiety and stress among communities and the HCWs, particularly in developing countries. The HCWs were at higher risk of developing stress and having poor wellbeing and mental health due to the long working hours, increased workloads, a shortage of personal protective equipment, the social stigma associated with COVID-19 infection, and lacked incentives to continue working [1].

Stress in the broad sense referred to any scenario that puts an individual under unusual physical or psychological strain and disrupts their equilibrium [2]. The World Health Organisation (WHO) defines stress as "any change that results in physical, emotional, or psychological strain due to body’s reaction to anything that demands attention or action " [3]. Before the COVID-19 pandemic, HCW stress levels were already above 60% for physicians, advanced care providers, and nurses [4].

The pandemic had resulted in the prevalence of a wide range of psychological problems such as fear, anxiety, stigma, prejudice, marginalisation towards the disease, and its relation to all people from healthy individuals and at-risk individuals to care workers [5]. Studies have shown that healthcare workers have experienced a range of unprecedented psychological stress on HCWs, such as anxiety, fear, panic attacks, post-traumatic stress symptoms, psychological distress, stigma, avoidance of contact, depressive tendencies, sleep disturbances, helplessness, interpersonal and isolation from family and social support, as well as concerns about their friends and family being exposed to infection [6]. A meta-analysis that systematically review the prevalence of anxiety and depression among frontline healthcare workers during the coronavirus disease 2019 (COVID-19) pandemic reported that the overall prevalence of anxiety and depression among frontline healthcare workers was 43% and 45%, respectively [7]. Meanwhile, a 2022 survey of 11,964 nurses found that over 70% reported recently experiencing stress and nearly 30% of 2,373 physicians reported high stress [4].

The high prevalence of psychological distress during the COVID-19 pandemic among the HCWs was reported to associated with many risk factors such as female sex, being a nurse, being young, living alone/being single, and having a chronic disease or psychiatric disorder history are the main risk factors at the personal level [8]. Meanwhile, having many years of work experience, the presence of COVID-19 symptoms and contact history, not enough sleep, having lower family support and limited social relationships, fear of infecting friends and family, having a reduced perception of protection by personal protective equipment, working on the frontline, and having longer service duration were found to be factors influencing the development of psychological distress during the COVID-19 pandemic [8]. Extended periods of high stress levels are a precursor to burnout which was defined by WHO [9] as prolonged stress that culminates in emotional exhaustion, depersonalization, and diminished professional efficacy. The present study was carried out with the aim of assessing the prevalence of stress, as well as to evaluate the risk factors that may lead to the development of stress among the healthcare workers.

## Materials and methods

### Study design, study population and sampling

This retrospective cross-sectional study was employing a universal sampling among 533 HCWs in Kota Setar District Health Office from a total of 958 available data. Stress was measured using the Depression Anxiety Depression Scale 21 (DASS-21), which was screened to all related HCWs between January and June 2021. The inclusion criteria were healthcare workers involved in managing COVID-19 at Kota Setar District Health Office. Written informed consent was obtained from all participants. Universal sampling was implemented.

### Data collection

The HCWs answered the DASS-21 through the Mental Health Online System. The scoring for the DASS-21 are as follows: normal (0–14), mild (15–18), moderate (19–25), severe (26–33) and very severe (34 and above). DASS-21 has been used extensively in many research including those conducted in Malaysia. It is a valid and highly reliable instrument with the Cronbach’s alpha value of 0.959, reflecting an excellent internal consistency. The Cronbach’s alpha value of each sub-scale for anxiety, depression, and stress were 0.87 (95% CI 0.86 to 0.89), 0.92 (95% CI 0.91 to 0.93) and 0.89 (95% CI 0.88 to 0.90) [10]. For the purpose of analysis, the stress score was categorized into two groups: Normal (Normal and mild stress) and Stress (moderate, severe, and very severe stress). Other information recorded in the online system were age, gender, ethnicity, education level, religion, job title, job designation, place of work and stress level. The relevant information was entered into the data collecting form after a review of all complete records that were accessible within the designated times.

### Statistical analysis

The data were analysed using the Statistical Package for Social Sciences (SPSS), version 25.0 Descriptive analysis was performed by using frequencies, percentage, mean, and standard deviation for normally distributed data to describe the characteristics of the respondents. Multiple logistic regression was conducted to measure the best model for predictors. Confidence interval was set at 95% and level of significance is set at 0.05.

Ethical approval had been obtained from the Medical Research and Ethics Committee (MREC) to carry out the research with NMRR registration: NMRR-21-361-58664. Permission to conduct the actual study in Kedah State Health Department was also obtained from the Director of Kedah State Health Department.

## Results

### Prevalence of stress and characteristics of respondents

Table 1 is showing the prevalence of stress among the HCWs included in this study, with majority did not experiencing stress (89.5%).

**Table 1:** Stress distribution.

Stress level	n (%)	95%Confidence Interval	
		Lower	Upper
Normal	477 (89.5)		
Stress	56 (10.5)	0.08	0.13

Meanwhile, the background characteristics of the respondents are described in Table 2. The median age of the respondents was 36 years of age with a minimum age of 23 and maximum age of 59. Female respondents comprised of 79.0% of the total respondents. Malay and Muslims (92.5%) were the dominated ethnic group and religion among the respondents and only 7.5% were Chinese, Indian and others. Three quarter of them (77.3%) had level education of diploma and below. The respondents were mostly working in the clinical related area which consist of 84.8% and 76.9% of the respondents were supporting staff.

**Table 2:** Distribution of respondents by sociodemographic and employment characteristics (N = 533).

Variables	Median (IQR)	n (%)
**Age (Years)**	**36.0 (26.0,46.0)**	
**Age Group**		
Below 40 years old		343 (64.4)
40 years old and above		190 (35.6)
**Gender**		
Male		112 (21.0)
Female		421 (79.0)
**Ethnicity**		
Malay		493 (92.5)
Others		40 (7.5)
**Religion**		
Muslim		493 (92.5)
Others		40 (7.5)
**Education level**		
Degree and above		121 (22.7)
Diploma and below		(77.3)
**Job title**		
Clinical		452 (84.8)
Non-clinical		81 (15.2)
**Place of work**		
Clinic		452 (84.8)
Health district office		81 (15.2)
**Job designation**		
Management and professional *(175)		123 (23.1)
Supporting staff *(794)		410 (76.9)

Note: a. management and professional consist of specialist, medical officer, dentist, pharmacist and social sciences officer.

Supporting consist of assistant environmental health officer/environmental health officer, radiographer, occupational therapist, technologist, nurses,vhealthcare assistant and administrative assistant.

b. Others religion consist of Buddha and Hindu.

### Factors associated with stress among HCWs

Tables 3 and 4 are showing the factors associated with stress among the respondents which were tested using the chi square test. Among the sociodemographic included in this study, only age (p-value = 0.019) and education level (p-value < 0.001) were significantly associated with stress among HCWs. Meanwhile, among the employment factors, place of work (p-value = 0.031) and job designation (p-value = 0.002) were significantly associated with stress among HCWs.

**Table 3:** The Chi-square test of stress (Normal vs Stress) of respondents by sociodemographic factors.

Variables	Stress		Test statistics		
			*X* ^2^	*df*	*P-value*
	Normal n(%)	Stress n(%)			
**Age**			5.515	1	0.019
Below 40 years old	299(87.2)	44(12.8)			
40 years old and above	178(93.7)	12(6.3)			
**Gender**			1.256	1	0.262
Men	97(86.6)	15(13.4)			
Women	380(90.3)	41(9.7)			
**Ethnicity**			0.183	1	0.669
Malay	442(89.7)	51(10.3)			
Others	35(87.5)	5(12.5)			
**Religion**			0.183	1	0.669
Muslim	442(89.7)	51(10.3)			
Non- Muslim	35(87.5)	5(12.5)			
**Education level**			12.033	1	0.001
Diploma and below	379(92.0)	33(8.0)			
Degree and above	98(81.0)	23(19.0)			

*Significant at P<0.05.

Note: Others religion consist of Buddha and Hindu.

**Table 4:** The Chi-square test of stress (Normal vs Stress) of respondents by employment factors.

Variables	Stress		Test statistics		
			*X* ^2^	*df*	*P-value*
	Normal n(%)	Stress n(%)			
**Job Title**			0.040	*1*	0.841
Clinical	404(89.4)	48(10.6)			
Non- Clinical	73(90.1)	8(9.9)			
**Place of Work**			4.666	*1*	0.031
Clinic	410(90.7)	42(9.3)			
Health district office	67(82.7)	14(17.3)			
**Job Designation**			9.261	*1*	0.002
Management and professional	101(82.1)	22(17.9)			
Supporting	376(91.7)	34(8.3)			

Note: a. management and professional consist of specialist, medical officer, dentist, pharmacist and social sciences officer.

Supporting consist of assistant environmental health officer/environmental health officer, radiographer, occupational therapist, technologist, nurses, healthcare assistant and administrative assistant.

b. Clinical work consists of those managing directly with the COVID-19 patients includes drivers, specialist, medical officer, dentist, pharmacist, radiographer, occupational therapist, nurses, and healthcare assistant.

### Predictors of stress

The multivariate logistic regression analysis revealed only place of work and education level were significantly predicting development of stress among the HCWs during the COVID-19 pandemic. Those who were working at the health district office had 2.3 (AOR = 2.310, 95% CI 1.177–4.535) increase likelihood to develop stress compared to those working at the clinics. Meanwhile, HCWs with degree and higher qualifications had 3 (AOR = 2.899, 95% CI 1.613–5.211) times higher chance of experiencing stress than personnel with only a diploma and below (Table 5).

**Table 5:** Predictors of stress among healthcare workers.

Variable	B	p-value	Adjusted Odds Ratio	95% Confidence interval	
				Lower	Upper
**Place of work** Health district office	0.837	.015*	2.310	1.177	4.535
**Education level** Degree and above	1.064	<0.001*	2.899	1.613	5.211
Constant	-2.624	<0.001	.072		

*Significant at p<0.05, clinic and Diploma and below as reference category.

## Discussion

The findings of this study shed light on the prevalence of stress among the HVWs and the associated factors. The prevalence reported in this study is lower compared to those previously reported in Trinidad and Tobago with 17.97% [11], 54.7% in India [12], 93.7% in Iraq [13] and 64.3% in Iran [14]. Nevertheless, the prevalence is almost the same with the study by Chew et al. [15] which were conducted among HCWs from five major hospitals involved in the care of COVID-19 patients in Singapore and India, with a prevalence of 5.2%, and another study among HCWs in Italy by Lenzo et al. [16] reported a prevalence of 8.9%.

The variation in the reported prevalence in various studies may have been contributed to the different time of stress measurement. As we know there were several waves of Covid-19 pandemic reflected by the different number of cases which can directly impacting the workload of the healthcare workers, as well as their stress levels. Furthermore, measuring stress at different stages of the pandemic may revealed different prevalence, which may closely related to the number of cases, support in place for the workers, better coping as well as work fatigue. Work fatigue (also referred to as exhaustion) is central to job burnout theories, such as the Job Demands-Resources (JDR) model and conservation of resources (COR) theory [17]. Work fatigue and burnout research during the pandemic has predominantly focused on specific occupations such as frontline healthcare workers and teachers as the pandemic has placed considerable psychological strain on members of these professions [17].

Nevertheless, some evidence have showed that while mental health symptoms were elevated among general populations in the early months of the pandemic, there is evidence of a normalisation of rates toward the end of 2020 and early 2021 both globally [18].This may represent a broad adaptation among the general public to the pandemic, and is in line with the expectation that the majority of people do not develop psychopathologies after natural disasters [19]. However, Individuals with a history of mental health treatment, loneliness, and lower resilience showed sustained or increased levels of mental health symptoms from March 2020 to April 2021, indicating a heterogenous psychological response to the pandemic that warrants further exploration [20].

### Factors predicting stress among HCWs during the COVID-19 pandemic

Place of work and education level were the only two factors found to significantly predict stress among the HCWs during the pandemic in this study. Working at the health district office exposed the HCW workers to a different level of stress. These HCWs at the district level comprise assistant environmental health officer/environmental health officer (AEHO/EHO), operation assistant, technologist, clerk, and administrative assistant. The stress and burden of work were obvious among the AEHO/EHO, who were tasked to manage and investigate each COVID-19 case reported to the health district office. The daily ever-increasing number of cases limits their time to manage and investigate the case for control measures. The AEHO explained that their source of occupational-related stress during the pandemic is due to the work burden associated with the high number of cases and poor management from their supervisor.

Among the roles and obligations of the EHOs during the COVID-19 pandemic were conducting screenings of travellers at the frontiers (air, land, and sea), investigating cases that have been reported or groups of recent incidents, locating individuals who had exposure to or interactions with suspected and confirmed cases, inspection of quarantine facilities (environmental health requirements), monitoring of individuals who are either self-quarantined or under observation at their residences, monitoring widespread disinfection in buildings and public spaces, overseeing the interments of all COVID-19 fatalities, upholding the conditions of lockdowns (Mass Movement Control Phases), monitoring food safety in food facilities and quarantine facilities, monitoring standard operating procedure compliance throughout the various Movement Control Order phases, implementation of the 1988 Prevention and Control of Infectious Disease Act’s requirements and to bring legal action against anyone who violate the COVID-19 provisions [21]. Therefore, they played crucial roles in emergency response duties, offer public information and guidance, uphold public health regulations, and to safeguard the public health during a public health emergency like a pandemic, which contributed towards the massive workload and development of stress during the pandemic. Furthermore, they also involved in fieldwork and frequent travel with long and irregular working hours.

On the other hand, highly educated people are more likely to hold better or higher positions in an organisation. They are responsible for many important tasks, which indicate more responsibilities and frequently involved in decision-making, that put them more at risk of occupational-related stress. The healthcare profession is a career that requires autonomous decision-making associated with patient care, technology, and the specialization of cases [22]. Healthcare decisions are often influenced by heavy time pressure and initial ambiguity when attempting to treat patients, hindering the ability to acquire necessary evidence for efficient patient care [23]. Unfortunately, uncertainty and ambiguity are commonplace within a healthcare setting which can interfere with making a proper medical decision.

Besides the above discussed issues, the issue of vaccine attitude among healthcare workers needs to be highlighted because it is crucial for the healthcare workers to keep healthy during the pandemic. According to a study by Rad et al., 32% of medical professionals are concerned about vaccinations, whereas 57.2% of them are confident in them [24]. Those who were not vaccinated showed much less fear of COVID-19 and trust in the healthcare system, which includes immunization. Being male, fearing COVID-19 more than others, and not having any underlying medical conditions are associated with a greater willingness to receive the COVID-19 vaccination [24]. The major causes of concern regarding vaccine reluctance are uncertainty about the vaccine’s efficacy, fear of getting sick, worry of contracting the disease after vaccination, fear of side effects, and the idea that vaccinations cause more harm than good. Both participants who were willing to get vaccinated and those who were not reported holding the beliefs that vaccine manufacturers are driven by profit and that vaccinations impair immunity [25]. HCWs may encountered a range of unfavourable, systemic, and localized circumstances. Fatigue and muscle pain following the first dose of COVID-19 vaccination were substantially correlated with sleep difficulties, worry and nervousness, distress and rage, despair and upset, inferiority to others, and suicidal thoughts [26]. Employee faith in medical care and vaccinations may increase if they perceive the healthcare system as a cooperative source of information when they make difficult health decisions on their own [25].

## Conclusions

The prevalence of stress reported in this study was considerably low. HCWs who were working at the district health office, as well as those with higher education level were more at risk of stress. The point of time when the data was collected during the pandemic will give better explanations on the prevalence of stress among the HCWs. Stress among workers in general and HCWs specifically is highly preventable through the presence of conducive working practices and policy, as well as the availability of effective support system. In the context of the COVID-19 pandemic, it is advised that health policies that track mental health and foster individual resilience be put into place. Additionally, risk factors for each population group should be identified in order to establish customized strategies [27].

## Supporting information

S1 File (XLSX)

S2 File (XLSX)
